# PASS2: update of database of structure-based sequence alignments

**DOI:** 10.1093/database/baaf072

**Published:** 2025-11-13

**Authors:** Revathy Menon, Soumya Nayak, Rama Rajesh, Ramanathan Sowdhamini

**Affiliations:** National Centre for Biological Sciences (Tata Institute of Fundamental Research), GKVK Campus, Bellary Road, Bangalore, Karnataka 560065, India; National Centre for Biological Sciences (Tata Institute of Fundamental Research), GKVK Campus, Bellary Road, Bangalore, Karnataka 560065, India; Institute for Stem Cell Science and Regenerative Medicine, GKVK Campus, Bellary Road, Bangalore, Karnataka 560065, India; National Centre for Biological Sciences (Tata Institute of Fundamental Research), GKVK Campus, Bellary Road, Bangalore, Karnataka 560065, India; Molecular Biophysics Unit, Indian Institute of Science, Mathikere, Bangalore, Karnataka 560012, India; Computational Biology, Institute of Bioinformatics and Applied Biotechnology, Electronic City, Bangalore, Karnataka 560100, India

## Abstract

Protein sequence alignments are evolutionary models and offer as starting points for the recognition of additional members of a homologous family and design of experiments. However, the accuracy of sequence alignments is obscured at the superfamily level due to distant relationships. Where structures of proteins are available, distantly related proteins can be aligned, guided by structural features. The Protein Alignment Organized as Structural Superfamilies (PASS2) database offers such structure-based sequence alignments for protein domains classified within superfamilies, as per the Structural Classification of Proteins extended (SCOPe) framework. The present update of PASS2 (PASS2.8) corresponds to the latest SCOPe release (version 2.08). This release comprises data for 26 690 protein domains exhibiting less than 40% sequence identity, organized into 2058 superfamilies. Several features derived from these alignments, including conserved secondary structural motifs, hidden Markov models (HMMs), conserved residues, and interactions across superfamilies, are also provided. For superfamilies containing divergent members, a k-means clustering algorithm has been employed to identify outliers and partition domains into split superfamilies. Novel features in this update include topological diagrams of the domains, potential interactors for each domain, and an updated methodology for identifying conserved interactions across superfamilies. This version of the database can be reached from http://caps.ncbs.res.in/pass2.

## Introduction

Comprehension of numerous biological mechanisms and processes is greatly aided by understanding the intricate relationship between sequence, structure, and function of proteins. However, significant sequence divergence, which can occur even among functionally related proteins, often limits the utility of conventional sequence alignment methods. Such divergence can obscure homologous relationships and hinder the identification of conserved functional elements. In these instances, structure-based alignment offers a more robust model, which is particularly effective for identifying conserved residues that are critical for maintaining protein function, even when sequence homology is low. These methods also facilitate the modelling of homologous proteins, which share a common evolutionary origin but may have diverged in functional roles.

The rapid growth of the Protein Data Bank (PDB; [1, 2]) has provided a substantial and continually expanding dataset that is invaluable for studying protein structure–function relationships. The Structural Classification of Proteins (SCOP; [3, 4]) database organizes proteins based on their structural and evolutionary relationships, using a combination of manual examination and automated computational methods to ensure accuracy and reliability. SCOP serves as an essential resource and facilitates ongoing research for comparative protein analysis, supporting structure prediction, identification of structural folds, evolutionary linkages, and functional annotation. SCOPe [5], an extended version of SCOP, periodically updates these classifications to incorporate newly discovered structures and provide enhanced resolution of evolutionary connections among proteins. These periodic updates by SCOPe are crucial for keeping abreast of the growth of PDB and support protein structural analyses.

The Protein Alignment Organized as Structural Superfamilies (PASS2) database contains alignments of protein domains within superfamilies, using data from the SCOPe database. Originally conceived as CAMPASS [6], the PASS2 algorithm has undergone several refinements, with releases corresponding to major updates of the SCOPe database, thereby maintaining the alignment accuracy and relevance [7–14]. In PASS2, domains exhibiting less than 40% sequence identity are selected specifically for structure-based alignments; those with greater sequence identity are excluded, as they can be effectively aligned using more conventional multiple sequence alignment tools like Clustal Omega [15] or MAFFT [16]. PASS2 provides additional resources such as detailed alignment annotations, statistical analysis of alignment quality, hidden Markov model (HMM) profiles [17], identification of highly conserved residues, and putative interactors of the domains.

Domains with distinctive structural features like large insertions [18, 19], deviant from other superfamily members, often deteriorate the alignment quality because of these unique elements. Such domains are referred to as ‘outliers’ [20] and are present in ∼6% of the total number of superfamilies considered in this update. These superfamilies often present problematic cases, presenting additional challenges to automatic updates. In the previous update, we had proposed an approach where recognition of outliers and re-alignment after splitting the superfamily are based on the k-means algorithm [8]. In this paper, apart from reporting the update, we present automation in dealing with outliers and also present this approach using detailed studies of four such superfamilies, one from each major structural class. The updated and eighth version of PASS2 database is available at http://caps.ncbs.res.in/pass2.

## Methods

**Figure 1.**
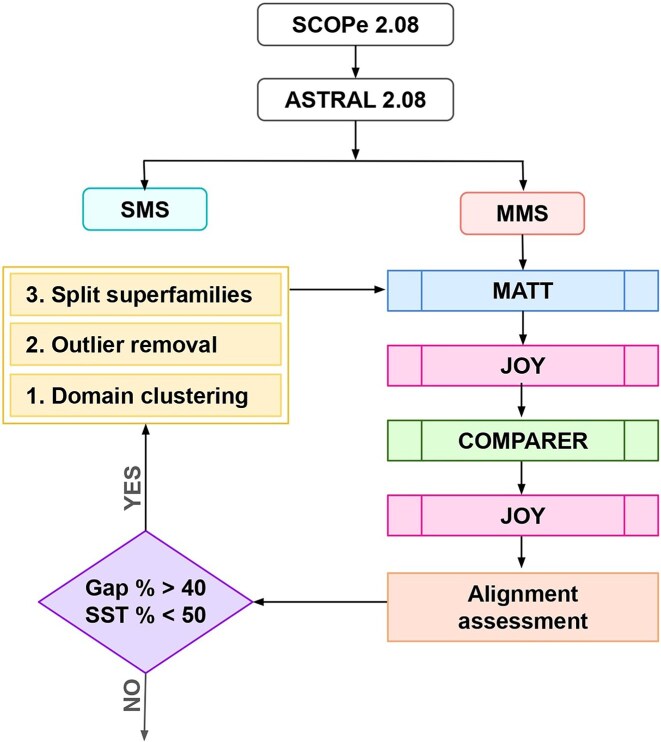
Workflow followed in PASS2.8. Superfamily alignments that do not meet the alignment assessment criteria undergo clustering of domains. The results of the clustering lead to ‘split superfamilies’, which are independently passed through the pipeline again. Domains identified as outliers with no parent cluster are removed from the superfamily before re-alignment.

The ASTRAL compendium within the SCOPe database provides PDB files of all protein domains within a superfamily. Data for superfamily members sharing less than 40% sequence identity were downloaded from ASTRAL [21], corresponding to SCOPe 2.08 [22], and organized according to SCOPe records. The dataset was then categorized into single-member superfamilies (superfamilies with a single member domain, hereafter referred to as SMSs) and multi-member superfamilies (superfamilies with more than one member domain, hereafter referred to as MMSs). An in-house Python script was used to remove heteroatoms, incomplete residues, and ambiguous and unknown residues from the domain PDB files. The programme Matt (Multiple Alignments with Translations and Twists; [23]) was used to perform an initial alignment, which was in turn used to derive aligned non-gapped sequence blocks, or equivalences, for all domains using JOY [24]. JOY was also used to annotate the initial alignment with structural features, including solvent accessibility, hydrophobicity, and hydrogen bonding. Based on structural dissimilarity among the superfamily domains, a tree was constructed and used as input for COMPARER [25] along with the initial Matt alignment. COMPARER is employed to produce a structure-guided sequence alignment using features such as secondary structure, hydrogen bonding, and solvent accessibility of amino acids. Equivalences were identified from the alignment and used for the rigid-body superposition of C^α^ backbones of domain structures with the MNYFIT programme within JOY [26]. The workflow is shown in Fig. 1.

In some cases, the final alignment may still contain a large number of gaps. This difficulty may arise when attempting to align one or more superfamily members that may be significantly different from the others. These differences can be brought about by structural dissimilarities (structural outliers) or large length variations between the superfamily domains (presence of dwarf or giant domains). This issue has been addressed by implementing a k-means algorithm, which is an unsupervised machine learning model, to cluster domains within such a superfamily [8].

The features used for clustering were the percentage of gaps for each domain in the alignment, the C^α^ mean root mean square deviation (RMSD) for each domain as calculated by JOY, and the length of the domain. K-means clustering was implemented in Python using the Scikit-learn library. The optimal number of clusters predicted and features used for the prediction were plotted for each superfamily, which enabled automatic recognition of outlier domains. The alignments were hence further improved either by splitting the superfamily into two or more split superfamilies based on the k-means clusters, or by removal of the outlier domains. The split superfamilies form structurally coherent subsets, and may be considered as structural subgroups. The alignment quality was assessed with the percentage of gaps for each domain in the final alignment, and the extent of conservation of secondary structure content (SST %) among the domains using ASSALIMAV, an in-house programme [12].

The alignment features for each superfamily include the construction of HMMs using the hmmbuild module from the HMMER suite [17]. The HMMs derived from these alignments enabled searches for newly deposited remote homologs within the SCOPe superfamily members in the PDB database [27]. Secondary structural motifs were characterized with Smotif [28], while alignment statistics were analysed with AliStat [29], and insertion-deletion (indel) information was assessed with CUSP [19]. To identify evolutionary conservation, residues with absolute conservation (100%) and high conservation (>90%) were identified across all superfamily members, designated as absolutely conserved residues (ACR) and highly conserved residues (HCR), respectively. Annotation of both alignments and PDB files was achieved through the JOY programme, adding structural features such as hydrogen bonding and solvent accessibility for each superfamily member. The average C^α^ RMSD-derived distance matrix, sequence identity matrix, mean C^α^ RMSD (in Å), and a structural dissimilarity tree have also been provided. For functional annotation, Gene Ontology (GO; [30]) terms were also associated with each superfamily member. Intra-domain interactions were identified with the aid of HORI, an in-house programme [31]. A pair of residues was considered as an interacting pair, if their C^α^ atoms were found to be within a distance of 7 Å. These interactions were then mapped back to the alignment to identify absolute conserved interactions, or interactions that are conserved amongst all domain members of a superfamily. The interactions are visualized for each individual domain wherever applicable, and are available for download as both image files and PyMol session files. Putative interactions are included for each superfamily member based on data from STRINGdb [32], and in this update, detailed topological diagrams of domain structures have also been provided using PDBsum1 [33].

The webserver is built using a combination of MariaDB 10.5 for database management and PHP 8 with the Smarty 5 templating engine for backend processing. Data preparation is handled using Python and BioPython, ensuring efficient organization and retrieval of biological datasets. The frontend is designed with HTML, CSS, Bootstrap, and jQuery to provide a responsive and user-friendly interface. Visualization components are implemented using Raphael and jsPhyloSVG for molecular structures and phylogenetic trees. Additionally, external databases and APIs such as SCOPe 2.08 and STRING-DB are integrated to dynamically fetch annotations and interaction data, enriching the analysis. Various Python scripts facilitate backend operations, including data parsing, processing, and visualization rendering.

## Results (and discussion)

### The PASS2.8 database

**Figure 2.**
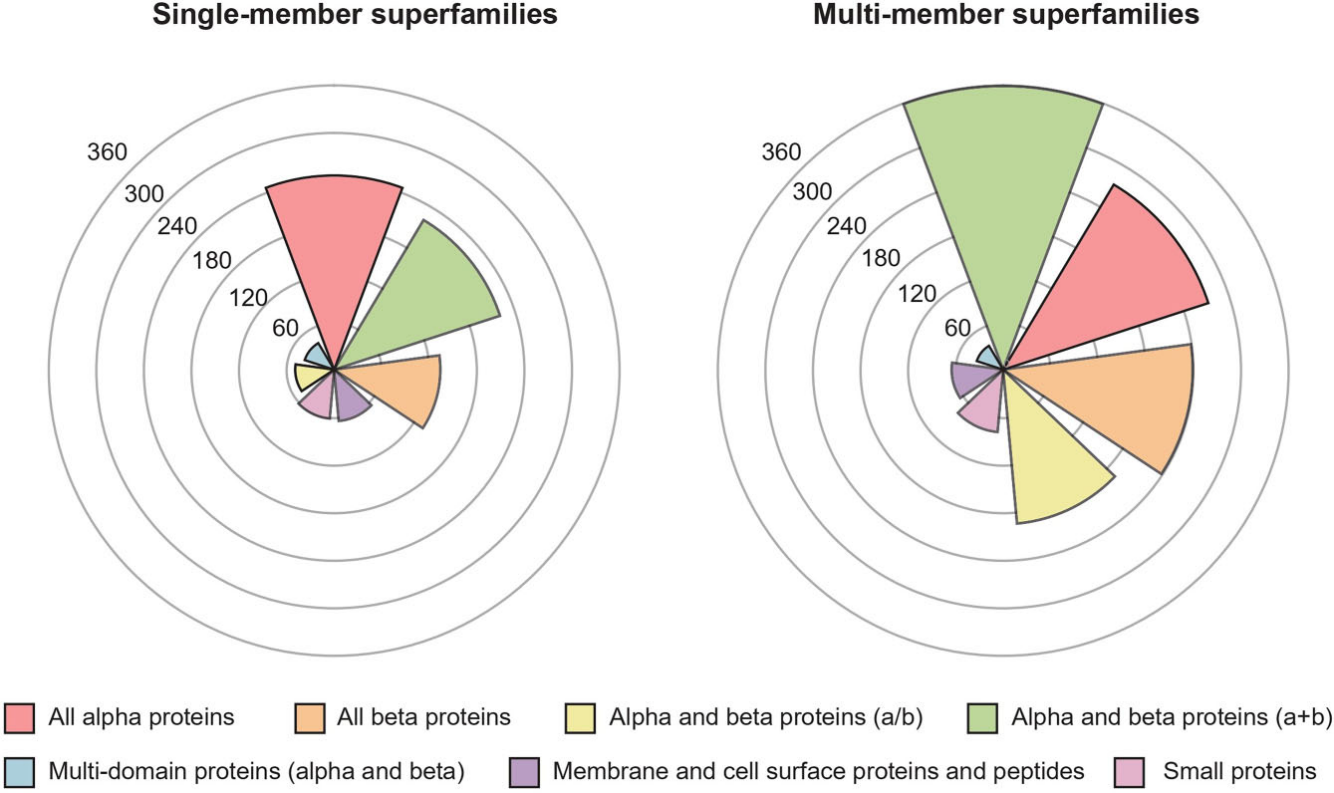
SCOPe class distribution of single-member superfamilies and multi-member superfamilies in PASS2.8. The all alpha ($\alpha$) class is the most represented class in single-member superfamilies, and the alpha and beta ($\alpha + \beta$) protein class is the most represented class in multi-member superfamilies.

In this latest version of the PASS2 database, a total of 2058 protein superfamilies have been taken into consideration, with 814 being single-member superfamilies (SMSs) and the remaining 1244 comprising multi-member superfamilies (MMSs). With the latest update to SCOPe, these superfamilies contain a total of 26 690 domains. SCOPe organizes all superfamilies into seven structural classes, and the number of superfamilies belonging to each class is represented in Fig. 2. In keeping with trends observed in previous versions of PASS2, the number of MMSs in the $\alpha$ and $\beta$ ($\alpha + \,\,\beta$) proteins class is the highest amongst all classes, and the same is true for the number of SMSs from the all-$\alpha$ class. The increase in the number of superfamilies as well as the number of domains in PASS2 over the years has been shown in Supplementary Fig. 1.

### Improving the structural alignment with protocol automation

The PASS2 alignment protocol comprises several steps, starting with data organization, preprocessing, followed by an initial alignment and annotation step, which is used to inform the final alignment, and the annotation of the final alignment (Fig. 1).

**Figure 3.**
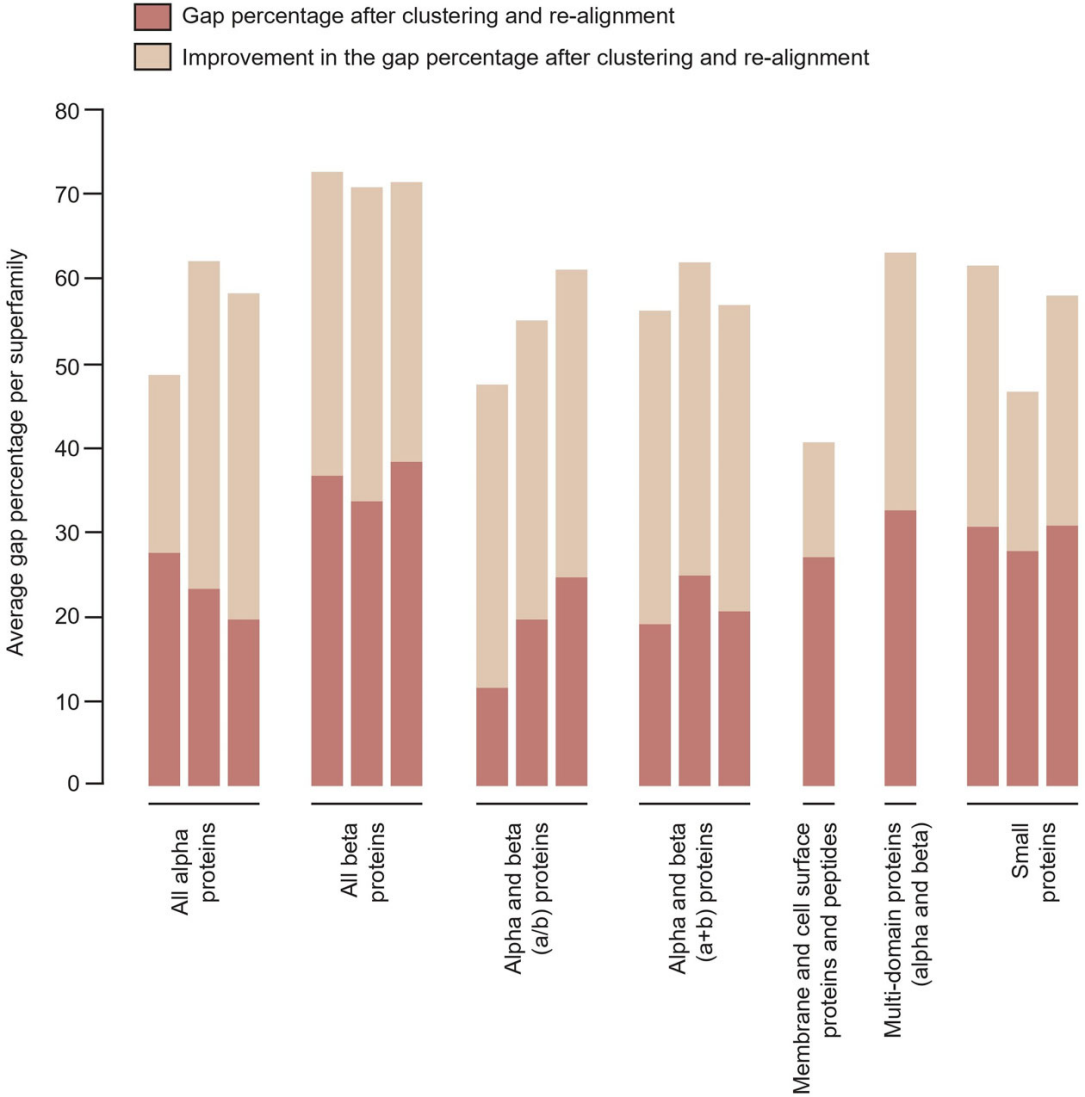
Plot representing the changes in the average gap percentage of the problematic cases presented by superfamilies. The superfamilies shown here are representative cases, and the full details are provided in Supplementary Table S1. The x-axis comprises superfamilies arranged by SCOPe class. The SCOPe IDs of the superfamilies here are, in order: (all $\alpha$ proteins class) 46 955, 47 781, and 48 600; (all $\beta$ proteins class) 49 785, 50 447, and 51 011; [$\alpha$ and $\beta$ proteins ($\alpha$/$\beta$) class] 54 189, 55 909, and 55 961; [$\alpha$ and $\beta$ proteins ($\alpha$+$\beta$) class] 53 223, 53 659, and 53 671; (membrane and cell surface proteins and peptides class) 56 935; [multidomain proteins ($\alpha$ and $\beta$)] 56 601; (small proteins) 57 501, 57 586, and 57903. The y-axis represents the average gap percentage in the alignments produced for each superfamily. The difference between the initial values for the average gap percentage and the improved values for the same is represented by the tan section of the bars. The improved values are in dark brown shade. A significant improvement can be observed before and after re-alignment. The sum of the difference and the improved values yields the original values of the average gap percentage.

In some cases, the final alignment may still contain a large number of gaps. This difficulty may arise when attempting to align one or more superfamily members that may be significantly different from the others. These differences can be brought about by structural dissimilarities or large length variations between the superfamily domains. After running the pipeline once, we identified 79 MMSs that had a gap per cent value more than 50%, as well as less than 40% conservation of secondary structure. These parameter thresholds were derived from an analysis of CAMPASS, a previous version of the database [34]. For these 79 superfamilies, k-means clustering was utilized as an objective and accurate method to identify the best way to improve the alignment. Based on the number of clusters obtained from this algorithm, each superfamily was split into smaller superfamilies, and the domains in these smaller or ‘split’ superfamilies were re-aligned with the PASS2 pipeline automatically. Figure 3 shows the improvement in the alignment of these superfamilies after the re-alignment for representative cases and the full list can be observed from Supplementary Table S1. If the clustering identified a domain outlier, either in terms of gap percentage or C^α^ RMSD, or both, the domain outlier was removed, and the remaining domains were re-aligned according to clusters obtained. In the case of domains with additional insertions, it is observed that removing such outliers improved the alignment rather than removing the insertions or ‘trimming’ the domain [13].

Cartoon diagrams of the domain structures made in PyMol, as well as topological diagrams generated using PDBsum1, are also provided. These files and PYMOL visualization script files are available for download. Absolutely and highly conserved residues within each superfamily are represented within the alignments, marked in red and blue colours, respectively. Absolutely conserved interactions (ACIs) identified for each superfamily are available as part of the database in the form of downloadable text files, png images, and PyMol session files. Potential domain interactions were identified using STRINGdb, where STRINGdb was queried for putative binding partners of each domain.

Other functional features provided within this version of the database include alignment statistics (AliStat), information regarding length variations caused by insertions or deletions (CUSP), conservation of secondary structural elements (ASSALIMAV), and structural motifs (SMotif). The structure-guided sequence alignments and the HMMs generated from them can be used to construct PSSMs, which may be used for sensitive sequence searches. More information about each member domain can be obtained such as GO terms and the dissimilarity tree. Structural features, *viz*. backbone conformation, hydrogen bonding patterns, and solvent-accessible surface area, are made available using JOY.

In PASS2.8, various steps in the PASS2 protocol have been automated, with programmes implemented to automatically detect, respond, and resolve errors in the protocol without requiring manual intervention. One example is the identification and removal of domain outliers in superfamilies. In-house Python scripts have been implemented to handle errors locally wherever required without disrupting the entire system. Repetitive tasks such as interaction identification and data extraction have been automated and exceptions are handled within the programme itself. Additionally, various logging frameworks have been implemented to standardize log entries for future reference. There is also documentation outlining common error scenarios, their potential causes, and recommended solutions. When combined, these steps reduce manual effort and time, and enhance accuracy in certain cases.

### Detailed study of outliers

This section covers 4 of the 79 superfamilies that presented as problematic cases for our pipeline, and required a second iteration through the pipeline to obtain an acceptable, adequate alignment.

#### SCOPe superfamily 52 777: CoA-dependent acyl transferases superfamily

**Figure 4.**
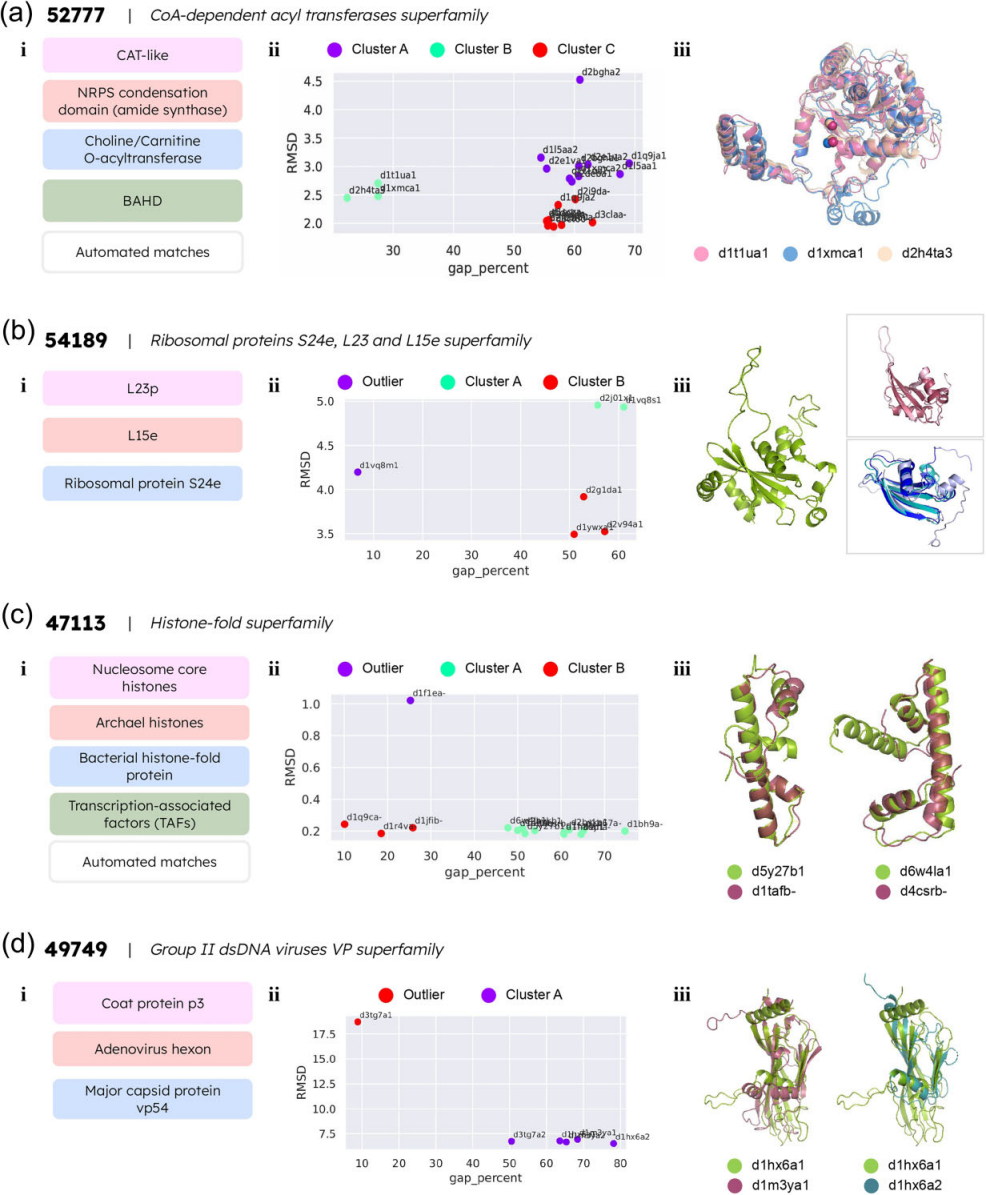
Detailed studies of four problematic superfamilies requiring re-alignment with the PASS2 protocol. (a) The CoA-dependent acyl transferases superfamily (i) contains four families and automatically matched domains without family annotations, termed as ‘automated matches’. (ii) The k-means clustering algorithm identified three clusters named A, B, and C. (iii) The automatically assigned domain d2h4ta3, and the domains d1t1ua1 and d1xmca1 from the choline/carnitine O-acyltransferase family were clustered together. The superimposed image of the three domains showcases their structural similarity. (b) The ribosomal proteins S24e, L23, and L15e superfamily has three families, shown in (i). (ii) The k-means clustering algorithm identified an outlier domain and divided the remaining domains into two clusters, A and B. (iii) The outlier domain d1vq8m1 is represented in green. The top panel on the right represents domains d2j01x1 (dark pink) and d1vq8s1 (light pink) from the L23p family, and the bottom panel represents domains d1ywxa1 (light violet), d2v94a1 (teal), and d2g1da1 (dark blue) from the ribosomal protein S24e family. (c) The histone-fold superfamily comprises four families and automatically matched domains (i). (ii) The k-means clustering identified one outlier domain, and two clusters named A and B. (iii) The figure represents the superimposed structures of the outliers with the respective closest clustered annotated domain. Left: domains d5y27b1 (green, automated match) and d1tafb_ (pink, TAFs family); right: d6w4la1 (green, automated match) and d4csrb_ (pink, TAFs family). (d) The group II dsDNA viruses VP superfamily consists of three families (i). (ii) The k-means algorithm identified one outlier domain, d3tg7a1. (iii) Length variations in viral proteins, especially in the loop regions, lead to clearer structural similarity between domain members of similar length from different families, as opposed to length-variant members of the same family. Left: superimposed structures of domains d1hx6a1 (green) and d1m3ya1 (pink) from the coat protein P3 and major capsid protein vp54 families, respectively; right: superimposed structures of domains d1hx6a1 (green) and d1hx6a2 (pink) from the coat protein P3 family.

The CoA-dependent acyl transferases superfamily in SCOPe comes under the $\alpha$ and $\beta$ ($\alpha$/$\beta$) proteins class, and belongs to the CoA-dependent acyl transferases fold. The superfamily is further divided into four families: the chloramphenicol acetyltransferase-like (CAT-like) family, the NRPS condensation domain (amide synthase) family, the choline/carnitine O-acyltransferase family, and the BAHD family (Fig. 4a(i)). With the exception of the CAT-like family, which has three member domains, all other families in this superfamily in our database have four member domains. Seven domains have been automatically matched to this superfamily, and have not been assigned families (Supplementary Fig. 2A). Domains in this superfamily generally have an architecture with two layers, containing both $\alpha$-helices and a mixed $\beta$-sheet.

The initial alignment exhibited a clear case of outlier retention (with an average alignment gap percentage of 54.8% and 16.8% conserved secondary structure) when assessed. As both parameter values are past the threshold for acceptable alignments, the superfamily was split into clusters based on the k-means algorithm as described in the *Methods* section. The results yielded three clusters with no clear outlier. Alignments were obtained for each of the clusters by treating each cluster as a ‘split superfamily’ and processing through the PASS2 pipeline. On constructing an RMSD-based tree for each split superfamily, it was observed that the automated matches were closely clustered with members from assigned families (Supplementary Fig. 2B). In clusters A and B, the d2deba1 domain and the d2h4ta3 domain, respectively, were found to cluster with members of the choline/carnitine O-acyltransferase family; in cluster C, the domains d2ii3a_, d3maea_, d6ct00_, d3l60a_, and d2i9da_ cluster with members from the CAT-like family (Fig. 4a(ii)). On further examining the clusters, it was found that this method permits the family-level annotation of the automatically matched domains. Figure 4a(iii) illustrates this point with the representation of the three structures from the second cluster: the automatically assigned domain d2h4ta3, and the domains d1t1ua1 and d1xmca1, both of which belong to the choline/carnitine O-acyltransferase family. Besides the apparent structural similarity between the three domains, the similar locations of the catalytic His residues, and the structurally important Asp residues located three residues apart in the three domains [35–37] also indicate a good family-level match. The structures of all domains are presented in Supplementary Fig. 2C. Hence, the results indicate that gap percentage and RMSD between domains are adequate to determine family annotations of automatically matched superfamily members.

#### SCOPe superfamily 54 189: ribosomal proteins S24e, L23, and L15e superfamily

The ribosomal proteins S24e, L23, and L15e superfamily in SCOPe is the only superfamily belonging to the eponymous fold, and is a part of the $\alpha$ and $\beta$ ($\alpha$+$\beta$) proteins class. This superfamily comprises three families: the L23p family with two domains, the L15e family containing one domain, and the ribosomal protein S24e family with three domains from our dataset (Fig. 4b(i); Supplementary Fig. 3A). It has no entries corresponding to SCOPe ‘automated matches’. Proteins belonging to this superfamily have a three-layer structure, with an antiparallel β-sheet sandwiched between two layers of α-helices.

The initial alignment revealed a 44.9% alignment gap percentage value and 20.2% conserved secondary structure. As these values do not fit the criteria for distinguishing acceptable alignments, the superfamily was split into clusters using the k-means algorithm as described earlier. The results yielded two clusters and an outlier, correctly grouping the six domains into their distinct families (Supplementary Fig. 3B). Cluster A contains the domains d2j01x1 and d1vq8s1 of the L23p family, while cluster B consists of the domains d2g1da1, d2v94a1, and d1ywxa1 from the ribosomal protein S24e family. The domain d1vq8m1 of the L15e family was recognized as an outlier (Fig. 4b(ii)). The structure of d1vq8m1 contains a loop insertion as shown in Fig. 4b(iii), which significantly increases its length (194 residues) compared to the other domains (81–102 residues) (see Supplementary Fig. 3C). The results obtained in this case indicate that gap percentage and RMSD between domains are important alignment and structural descriptors to distinguish family-level similarities and differences.

#### SCOPe superfamily 47 113: histone-fold superfamily

The histone-fold superfamily in SCOPe is the only member of the histone fold, and is classified as part of the all-$\alpha$ proteins structural class. This superfamily can be further classified into four families, namely the nucleosome core histones family, the archeal histones family (two domains), the bacterial histone-fold family (one domain), and the transcription-associated factors (TAFs) family (10 domains) (Supplementary Fig. 4A). Our dataset does not contain domains from the nucleosome core histones family owing to the 40% sequence similarity cut-off applied for curation, and it includes two domains that are automated matches (Fig. 4c(i)). The domains of this superfamily have three or more α-helices, with a long middle helix flanked by shorter helices at each terminal.

Assessment of the initial alignment displayed an alignment gap percentage value of 48.1% and 39.1% conserved secondary structures amongst the member domains. As these values do not meet the criteria set to identify satisfactory alignments, the k-means algorithm was applied. This resulted in the superfamily being split into two clusters, leaving behind an outlier domain (Fig. 4c(ii); Supplementary Fig. 4B). The domain d1f1ea_ was identified as an outlier, presumably due to the domain being 133 residues long, which is more than double the length of the other domain from the family in our dataset: the domain d1b67a_, which is 62 residues long. Cluster A contains the domain d1b67a_ from the archaeal histones family; the domains d1bh9a_, d1bh9b_, d1h3ob1, d1jfia_, d1tafb_, d2byka1, d2bykb1, and d4csrb_ from the TAFs family; and the automated matches d5y27b1 and d6w4la1. Cluster B consists of the domain d1r4va_ from the bacterial histone-fold protein family, and the domains d1jfib_ and d1q9ca_ from the TAFs family.

Domain d5y27b1 is part of the DNA polymerase epsilon subunit C, is also known as dpb4, and forms an H2A–H2B-like heterodimer with the protein dpb3. It is involved in DNA replication, transcription, and transcription regulation [38]. From this information, it seems likely that this domain should be a part of the TAFs family. Moreover, the dpb3–dpb4 heterodimer plays a vital role in coordinating the inheritance of histone post-translational modifications such as hypoacetylation and H3K9 methylation during replication [38]. Our results show that the domain clusters most closely with d1atfb_, a member of the TAFs family [39] (Fig. 4c(iii)). The topological comparison of the two domains is shown in Supplementary Fig. 4C.

Domain d6w4la1 is the H2B segment of a single-chain H2B–H2A histone chimera from *Xenopus laevis*, which is nearly identical to nucleosomal H2A/H2B [40]. As such, it has very high structural similarity to nucleosomal H2B. However, the nucleosomal core histones family is not part of our dataset. Amongst the remaining three families in the histone-fold superfamily, the TAFs family appears to be structurally closest to the nucleosomal core histones. The other two families are specific to archaeal and bacterial proteins, and so are distinguished from eukaryotic protein families. Hence, the TAFs family appears to be the best fit for domain d6w4la1 given our dataset. In our results, this domain is clustered most closely with the domain d4csrb_ (Fig. 4c(iii)), which is the NF-Y protein in *Arabidopsis thaliana*, and which forms a histone-fold heterodimer also similar to H2A–H2B [41, 42]. The structural similarities may be better appreciated *via* the topological diagrams of the domains, as shown in Supplementary Fig. 4C. Finally, the results for the domains d5y27b1 and d6w4la1 further validate that our k-means approach allows for the family-level annotation of automatically matched domains.

#### SCOPe superfamily 49 749: group II dsDNA viruses VP superfamily

The group II dsDNA viruses VP (viral protein) superfamily is one of seven superfamilies belonging to the nucleoplasmin-like/VP (viral coat and capsid proteins) fold, under the all-$\beta$ proteins class. The superfamily contains three families: the coat protein p3 family with domains d1hx6a1 and d1hx6a2, the adenovirus hexon family with domains d3tg7a1 and d3tg7a2, and the major capsid protein vp54 family with domains d1m3ya1 and d1m3ya2 (Fig. 4d(i); Supplementary Fig. 5A). There are no automated matches, and the general structure of domains belonging to this superfamily resembles a jelly roll fold, with two antiparallel $\beta$-sheets forming a sandwich-like structure.

The initial alignment assessment revealed a gap percentage of 55.8% and a conservation of secondary structures among member domains at 17.5%. Since these values did not meet the predefined criteria for satisfactory alignments, the k-means clustering algorithm was subsequently employed. This resulted in the identification of an outlier domain, d3tg7a1, from the adenovirus hexon family of proteins (Fig. 4d(ii); Supplementary Fig. 5B). This is likely due to long insertions in the loop regions in the domain structure, characteristic of adenovirus hexon proteins [43].

In this case, we attempted to improve upon the alignment in two different approaches and compare the results. The first approach involves truncating the obvious terminal insertions in the identified outlier before re-aligning, and the second one removes the outlier domain from the superfamily before re-alignment. In the first approach, removal of insertions from the terminals of the domain did not yield a satisfactory alignment owing to the presence of more insertions in the loop regions between the helices and strands, which resulted in gappy alignments (Supplementary Fig. 5C). Hence, the second one was followed as in the other cases, where the identified outlier domain was removed from the superfamily before re-aligning. In this case, domains from the major capsid protein vp54 family and the coat protein p3 family clustered together (Supplementary Fig. 5B), despite the coat protein p3 family being structurally similar (and possibly evolutionarily related) to the adenovirus hexon family [44]. This could be due to the length variations in the viral proteins (Supplementary Fig. 5D), as the adenovirus hexon family proteins are known to have long insertions in loop regions [43]. The subclusters formed by domains d1m3ya1 and d1hx6a1, and by domains d1m3ya2 and d1hx6a2, could be due to the length difference between d1hx6a1 and d1hx6a2—the difference in their lengths produces a poorer alignment than if d1hx6a1 were aligned with d1m3ya1 (Fig. 4d(iii)).

## Conclusions

The structure-based sequence alignments of 2058 protein domain superfamilies provide a foundational resource for advanced bioinformatic analyses. These alignments serve as robust evolutionary models, facilitating effective comparison of novel members and enabling researchers to design targeted biochemical experiments aimed at probing specific functional hypotheses. They also support the detailed examination of structurally conserved or variable regions, such as insertions and deletions, which are crucial for understanding functional adaptations and evolutionary divergence. The integration of accessory structural data, including conserved structural blocks, structure-annotated derived files, and networks of conserved residues, organized within the PASS2 database, enhances the potential for extensive structural and biochemical analyses. Supplementary Fig. 6 highlights two key aspects of our database: (A) the frequency distribution of mean RMSD values across split superfamilies and (B) the distribution of the number of clusters identified among the 79 structurally divergent superfamilies, reflecting the structural heterogeneity captured in PASS2.8. These data inform that the resolution of structural subgroups indeed reduces the mean RMSD values (mostly less than 4Ǻ) and to one or two clusters. The incorporation of machine learning techniques for identifying structural outliers is poised to streamline future updates automatically. This approach ensures that the alignments remain accurate, relevant, and reflective of the latest available data.

## Conflict of interest

None declared.

## Supplementary Material

baaf072_Supplemental_Files

## Data Availability

All superfamily data (including alignments, HMM profiles, conservation information, topological diagrams and superimposed structures) are accessible and downloadable via the PASS2.8 web portal at https://caps.ncbs.res.in/pass2/. SCOPe v2.08 member sequences are available for download from the SCOPe website (https://scop.berkeley.edu/downloads/).

## References

[1] Berman HM, Westbrook J, Feng Z et al. The Protein Data Bank. Nucleic Acids Res. 2000;28:235–42. 10.1093/nar/28.1.235 10592235 PMC102472

[2] Bernstein FC, Koetzle TF, Williams GJ et al. The Protein Data Bank: a computer-based archival file for macromolecular structures. J Mol Biol. 1977;112:535–42. 10.1016/S0022-2836(77)80200-3 875032

[3] Andreeva A, Kulesha E, Gough J et al. The SCOP database in 2020: expanded classification of representative family and superfamily domains of known protein structures. Nucleic Acids Res. 2020;48:D376–82. 10.1093/nar/gkz1064 31724711 PMC7139981

[4] Murzin AG, Brenner SE, Hubbard T et al. SCOP: a structural classification of proteins database for the investigation of sequences and structures. J Mol Biol. 1995;247:536–40. 10.1016/S0022-2836(05)80134-2 7723011

[5] Fox NK, Brenner SE, Chandonia J-M. SCOPe: structural classification of proteins—extended, integrating SCOP and ASTRAL data and classification of new structures. Nucleic Acids Res. 2014;42:D304–9. 10.1093/nar/gkt1240 24304899 PMC3965108

[6] Sowdhamini R, Burke DF, Huang JF et al. CAMPASS: a database of structurally aligned protein superfamilies. Structure. 1998;6:1087–94. 10.1016/S0969-2126(98)00110-5 9753697

[7] Bhaduri A, Pugalenthi G, Sowdhamini R. PASS2: an automated database of protein alignments organised as structural superfamilies. BMC Bioinf. 2004;5:35. 10.1186/1471-2105-5-35 PMC407847 15059245

[8] Bhattacharyya T, Nayak S, Goswami S et al. PASS2.7: a database containing structure-based sequence alignments and associated features of protein domain superfamilies from SCOPe. Database (Oxford). 2022;2022:baac025. 10.1093/database/baac025 35411388 PMC9216583

[9] Gandhimathi A, Ghosh P, Hariharaputran S et al. PASS2 database for the structure-based sequence alignment of distantly related SCOP domain superfamilies: update to version 5 and added features. Nucleic Acids Res. 2016;44:D410–14. 10.1093/nar/gkv1205 26553811 PMC4702857

[10] Gandhimathi A, Nair AG, Sowdhamini R. PASS2 version 4: an update to the database of structure-based sequence alignments of structural domain superfamilies. Nucleic Acids Res. 2012;40:D531–34. 10.1093/nar/gkr1096 22123743 PMC3245109

[11] Ghosh P, Bhattacharyya T, Mathew OK et al. PASS2 version 6: a database of structure-based sequence alignments of protein domain superfamilies in accordance with SCOPe. Database (Oxford). 2019;2019:baz028. 10.1093/database/baz028 30820573 PMC6395796

[12] Kalaimathy S, Sowdhamini R, Kanagarajadurai K. Critical assessment of structure-based sequence alignment methods at distant relationships. Briefings Bioinf. 2011;12:163–75. 10.1093/bib/bbq025 21422071

[13] Kanagarajadurai K, Kalaimathy S, Nagarajan P et al. A bioinformatics protocol for rigorous structure-based sequence alignment of distantly related proteins. Nat Protoc. 2009. 10.1038/nprot.2009.166

[14] Mallika V, Bhaduri A, Sowdhamini R. PASS2: a semi-automated database of protein alignments organised as structural superfamilies. Nucleic Acids Res. 2002;30:284–88. 10.1093/nar/30.1.284 11752316 PMC99156

[15] Sievers F, Higgins DG. Clustal Omega for making accurate alignments of many protein sequences. Protein Sci. 2018;27:135–45. 10.1002/pro.3290 28884485 PMC5734385

[16] Katoh K, Rozewicki J, Yamada KD. MAFFT online service: multiple sequence alignment, interactive sequence choice and visualization. Briefings Bioinf. 2019;20:1160–66. 10.1093/bib/bbx108 PMC6781576 28968734

[17] Eddy SR . Accelerated profile HMM searches. PLoS Comput Biol. 2011;7:e1002195. 10.1371/journal.pcbi.1002195 22039361 PMC3197634

[18] Mutt E, Mathew OK, Sowdhamini R. LenVarDB: database of length-variant protein domains. Nucleic Acids Res. 2014;42:D246–50. 10.1093/nar/gkt1014 24194591 PMC3964994

[19] Sandhya S, Pankaj B, Govind MK et al. CUSP: an algorithm to distinguish structurally conserved and unconserved regions in protein domain alignments and its application in the study of large length variations. BMC Struct Biol. 2008;8:28. 10.1186/1472-6807-8-28 18513436 PMC2423364

[20] Arumugam G, Nair AG, Hariharaputran S et al. Rebelling for a reason: protein structural ‘outliers’. PLoS One. 2013;8:e74416. 10.1371/journal.pone.0074416 24073209 PMC3779223

[21] Chandonia J-M, Hon G, Walker NS et al. The ASTRAL compendium in 2004. Nucleic Acids Res. 2004;32:D189–92. 10.1093/nar/gkh034 14681391 PMC308768

[22] Chandonia J-M, Guan L, Lin S et al. SCOPe: improvements to the structural classification of proteins—extended database to facilitate variant interpretation and machine learning. Nucleic Acids Res. 2022;50:D553–59. 10.1093/nar/gkab1054 34850923 PMC8728185

[23] Menke M, Berger B, Cowen L. Matt: local flexibility aids protein multiple structure alignment. PLoS Comput Biol. 2008;4:e10. 10.1371/journal.pcbi.0040010 18193941 PMC2186361

[24] Mizuguchi K, Deane CM, Blundell TL et al. JOY: protein sequence-structure representation and analysis. Bioinformatics. 1998;14:617–23. 9730927 10.1093/bioinformatics/14.7.617

[25] Sali A, Blundell TL. Definition of general topological equivalence in protein structures. A procedure involving comparison of properties and relationships through simulated annealing and dynamic programming. J Mol Biol. 1990;212:403–28. 2181150 10.1016/0022-2836(90)90134-8

[26] Sutcliffe MJ, Haneef I, Carney D et al. Knowledge based modelling of homologous proteins, Part I: three-dimensional frameworks derived from the simultaneous superposition of multiple structures. Protein Eng. 1987;1:377–84. 10.1093/protein/1.5.377 3508286

[27] Baldi P, Chauvin Y, Hunkapiller T et al. Hidden Markov models of biological primary sequence information. Proc Nat Acad Sci USA. 1994;91:1059–63. 10.1073/pnas.91.3.1059 8302831 PMC521453

[28] Pugalenthi G, Suganthan PN, Sowdhamini R et al. SMotif: a server for structural motifs in proteins. Bioinformatics. 2007;23:637–38. 17237055 10.1093/bioinformatics/btl679

[29] Wong TKF, Kalyaanamoorthy S, Meusemann K et al. A minimum reporting standard for multiple sequence alignments. NAR Genom Bioinforms. 2020;2:lqaa024. 10.1093/nargab/lqaa024 PMC7671350 33575581

[30] Gene Ontology Consortium, Blake JA, Dolan M, Drabkin H et al. Gene Ontology annotations and resources. Nucleic Acids Res. 2013;41:D530–35. 23161678 10.1093/nar/gks1050 PMC3531070

[31] Sundaramurthy P, Shameer K, Sreenivasan R et al. HORI: a web server to compute Higher Order Residue Interactions in protein structures. BMC Bioinf. 2010;11(Suppl 1):S24. 10.1186/1471-2105-11-S1-S24 PMC3009495 20122196

[32] Szklarczyk D, Nastou K, Koutrouli M et al. The STRING database in 2025: protein networks with directionality of regulation. Nucleic Acids Res. 2024;53:D730–37. 10.1093/nar/gkae1113 PMC11701646 39558183

[33] Laskowski RA . PDBsum1: a standalone program for generating PDBsum analyses. Protein Sci. 2022;31:e4473. 10.1002/pro.4473 36251626 PMC9667822

[34] Chakrabarti S, Sowdhamini R. Regions of minimal structural variation among members of protein domain superfamilies: application to remote homology detection and modelling using distant relationships. FEBS Lett. 2004;569:31–36. 10.1016/j.febslet.2004.05.028 15225604

[35] Govindasamy L, Pedersen B, Lian W et al. Structural insights and functional implications of choline acetyltransferase. J Struct Biol. 2004;148:226–35. 10.1016/j.jsb.2004.06.005 15477102

[36] Hsiao Y-S, Jogl G, Esser V et al. Crystal structure of rat carnitine palmitoyltransferase II (CPT-II). Biochem Biophys Res Commun. 2006;346:974–80. 10.1016/j.bbrc.2006.06.006 16781677 PMC2937350

[37] Jogl G, Hsiao Y-S, Tong L. Crystal structure of mouse carnitine octanoyltransferase and molecular determinants of substrate selectivity. J Biol Chem. 2005;280:738–44. 10.1074/jbc.M409894200 15492013

[38] He H, Li Y, Dong Q et al. Coordinated regulation of heterochromatin inheritance by Dpb3-Dpb4 complex. Proc Nat Acad Sci USA. 2017;114:12524–29. 10.1073/pnas.1712961114 29109278 PMC5703312

[39] Xie X, Kokubo T, Cohen SL et al. Structural similarity between TAFs and the heterotetrameric core of the histone octamer. Nature. 1996;380:316–22. 10.1038/380316a0 8598927

[40] Warren C, Bonanno JB, Almo SC et al. Structure of a single-chain H2A/H2B dimer. Acta Crystallogr F Struct Biol Commun. 2020;76:194–98. 10.1107/S2053230X20004604 32356520 PMC7193513

[41] Gnesutta N, Saad D, Chaves-Sanjuan A et al. Crystal structure of the *Arabidopsis thaliana* L1L/NF-YC3 histone-fold dimer reveals specificities of the LEC1 family of NF-Y subunits in plants. Mol Plant. 2017;10:645–48. 10.1016/j.molp.2016.11.006 27871811

[42] Romier C, Cocchiarella F, Mantovani R et al. The NF-YB/NF-YC structure gives insight into DNA binding and transcription regulation by CCAAT factor NF-Y. J Biol Chem. 2003;278:1336–45. 10.1074/jbc.M209635200 12401788

[43] Benson SD, Bamford JK, Bamford DH et al. Viral evolution revealed by bacteriophage PRD1 and human adenovirus coat protein structures. Cell. 1999;98:825–33. 10.1016/S0092-8674(00)81516-0 10499799

[44] Benson SD, Bamford JKH, Bamford DH et al. The X-ray crystal structure of P3, the major coat protein of the lipid-containing bacteriophage PRD1, at 1.65 A resolution. Acta Crystallogr D Biol Crystallogr. 2002;58:39–59. 10.1107/S0907444901017279 11752778

