# Supplementary material for: PASS2: update of database of structure-based sequence alignments
Source: Database (Oxford). 2025 Nov 13;2025:baaf072. doi: 10.1093/database/baaf072 (PMC12612674; doi:10.1093/database/baaf072)
Supplement: baaf072_Supplemental_Files [file baaf072_supplemental_files.zip › PASS2.8_Suppl_figures.pdf]

Supplementary File 1

Supplementary Figure 1

	PASS2 (2002)	PASS2.2 (2004)	PASS2.3 (2011)	PASS2.4 (2012)	PASS2.5 (2016)	PASS2.6 (2019)	PASS2.7 (2022)	PASS2.8
**SMS**	613	566	733	864	812	800	805	814
**TMS**	-	-	327	366	359	-	-	-
**MMS**	110	628	676	731	806	1206	1219	1244
**Total**	*723*	*1194*	*1776*	*1961*	*1977*	*2006*	*2024*	*2058*

There are 68620 more domains in SCOPe 2.08 compared to SCOPe 2.07.

PASS2.8 has 26690 domains; PASS2.7 had 14323 domains.

Supplementary Figure 1. *Table representing the changes in the number of single-member superfamilies (SMS), two-member superfamilies (TMS), and multi-member superfamilies (MMS) over versions of PASS2. Each version of PASS2 is in correspondence with a major update to SCOP, later SCOPe.*

Supplementary Figure 2

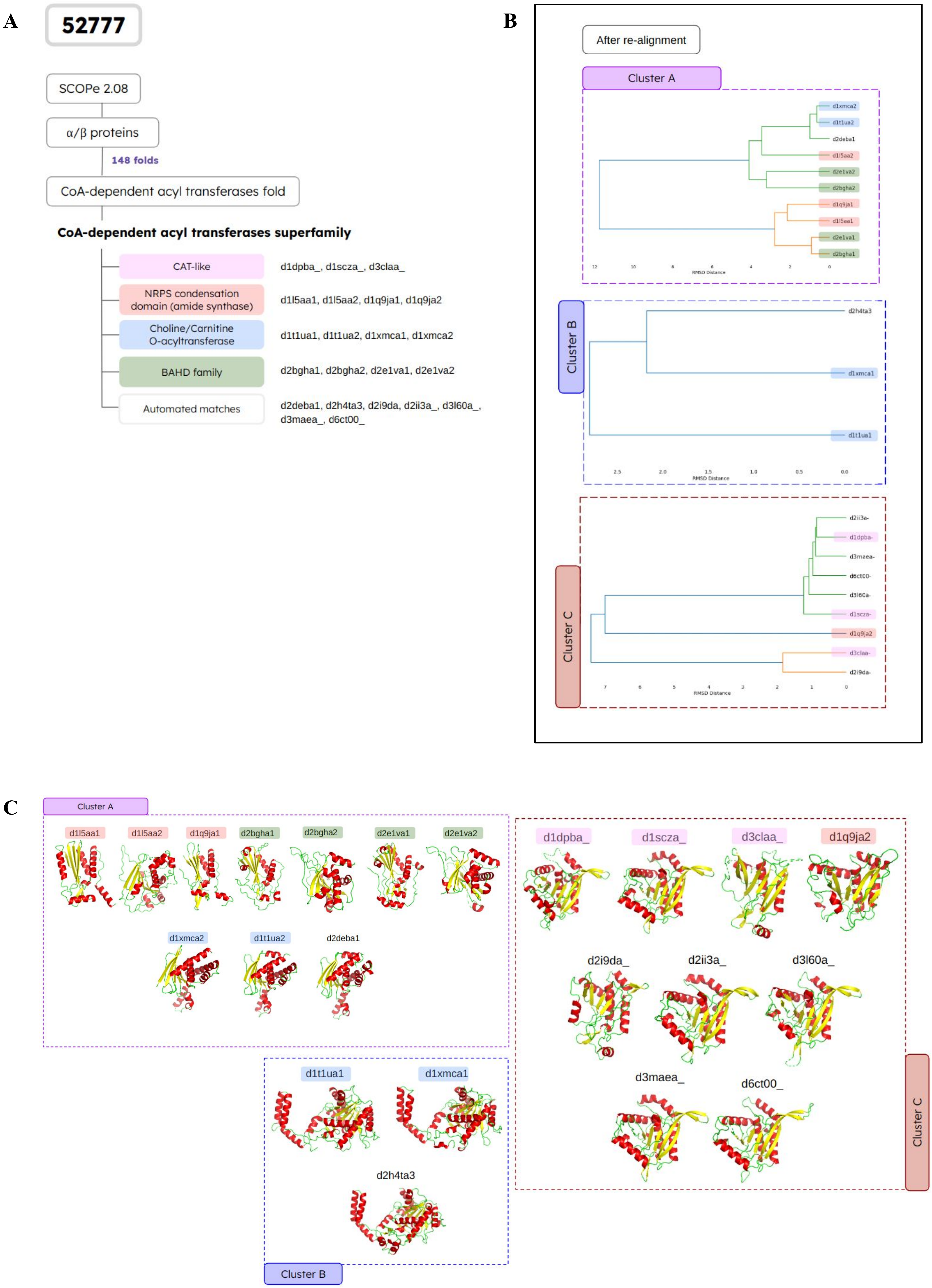

Supplementary Figure 2. *SCOPe superfamily 52777: CoA-dependent acyl transferases superfamily.* ***(A)*** *Domain classification according to SCOPe 2.08. The colour coding for the families presented here is followed all through the figure panels.* ***(B)*** *RMSD-based dendrogram generated after the first round of alignment.* ***(C)*** *RMSD-based dendrogram of the domains after being divided into clusters and re-aligned. Note that there are no outliers detected.* ***(D)*** *The PDB structures of the domains, labels coloured by family, grouped based on the results of the k-means clustering. Structures were visualised in PyMol.*

Supplementary Figure 3

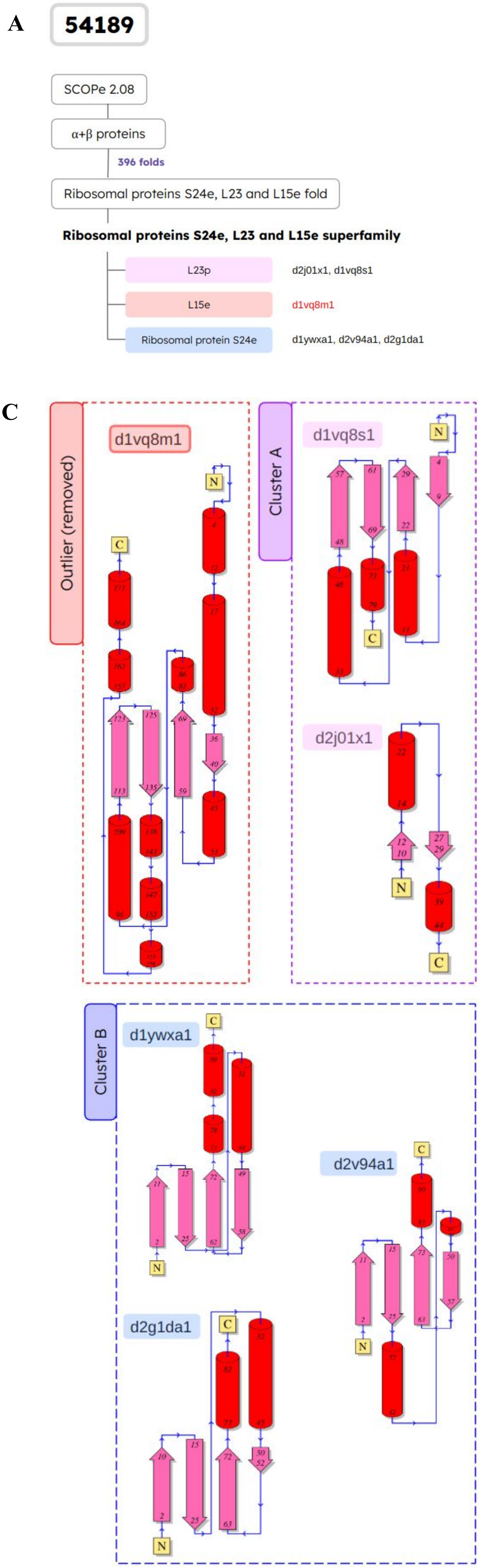

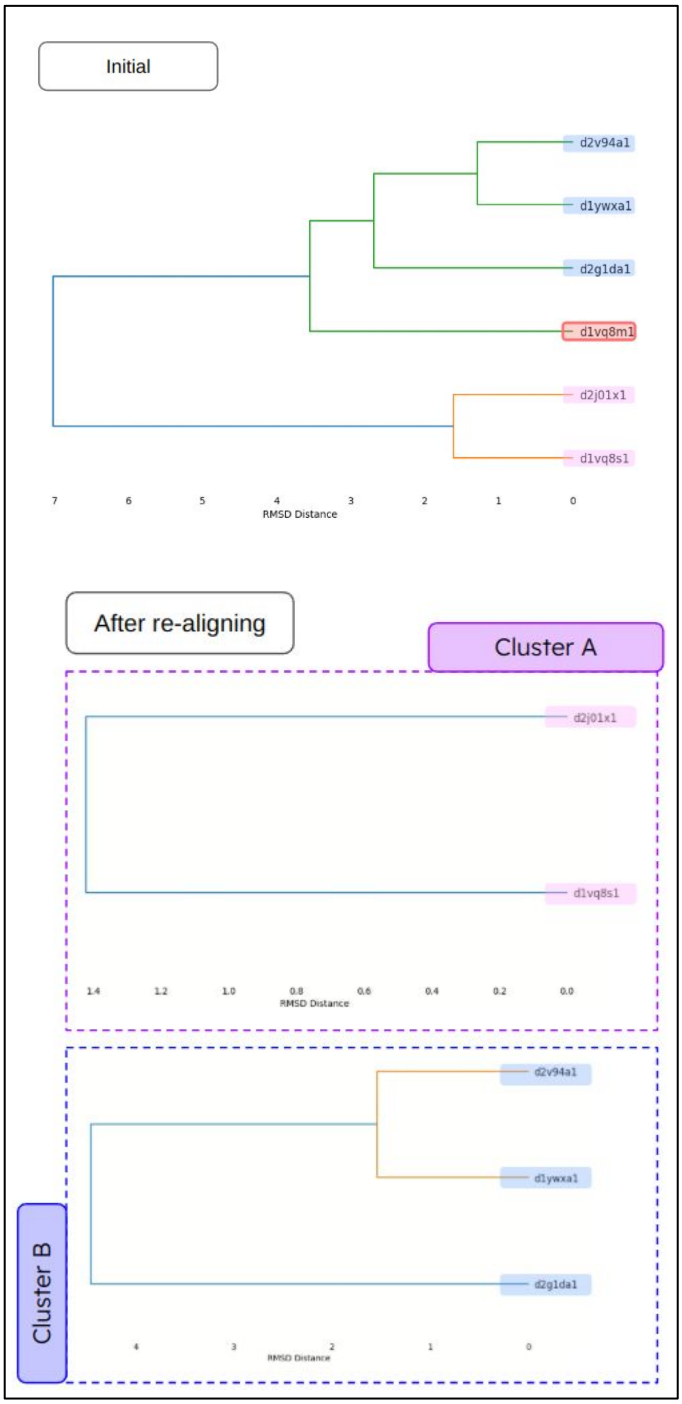

Supplementary Figure 3.
SCOPe superfamily 54189: Ribosomal proteins S24e, L23 and L15e superfamily. ***(A)*** *Domain classification according to SCOPe 2.08. The colour coding for the families presented here is followed all through the figure panels. The outlier is presented in read text, and wherever there is a box around the outlier label, the box has a red border.* ***(B)*** *RMSD-based dendrogram generated initially, and after the domains are divided into clusters and re-aligned. Note that the outlier is removed for re-alignment.* ***(C)*** *The topological diagrams of the domains generated using PDBsum1, labels coloured by family, grouped based on the results of the k-means clustering.*

Supplementary Figure 4

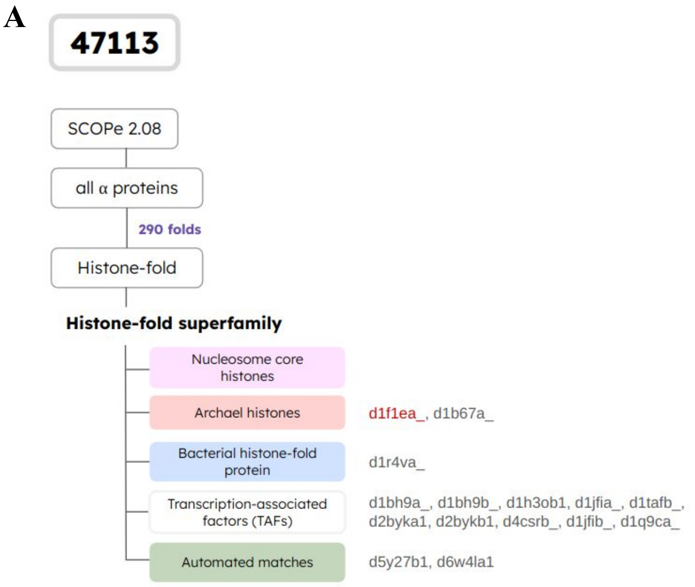

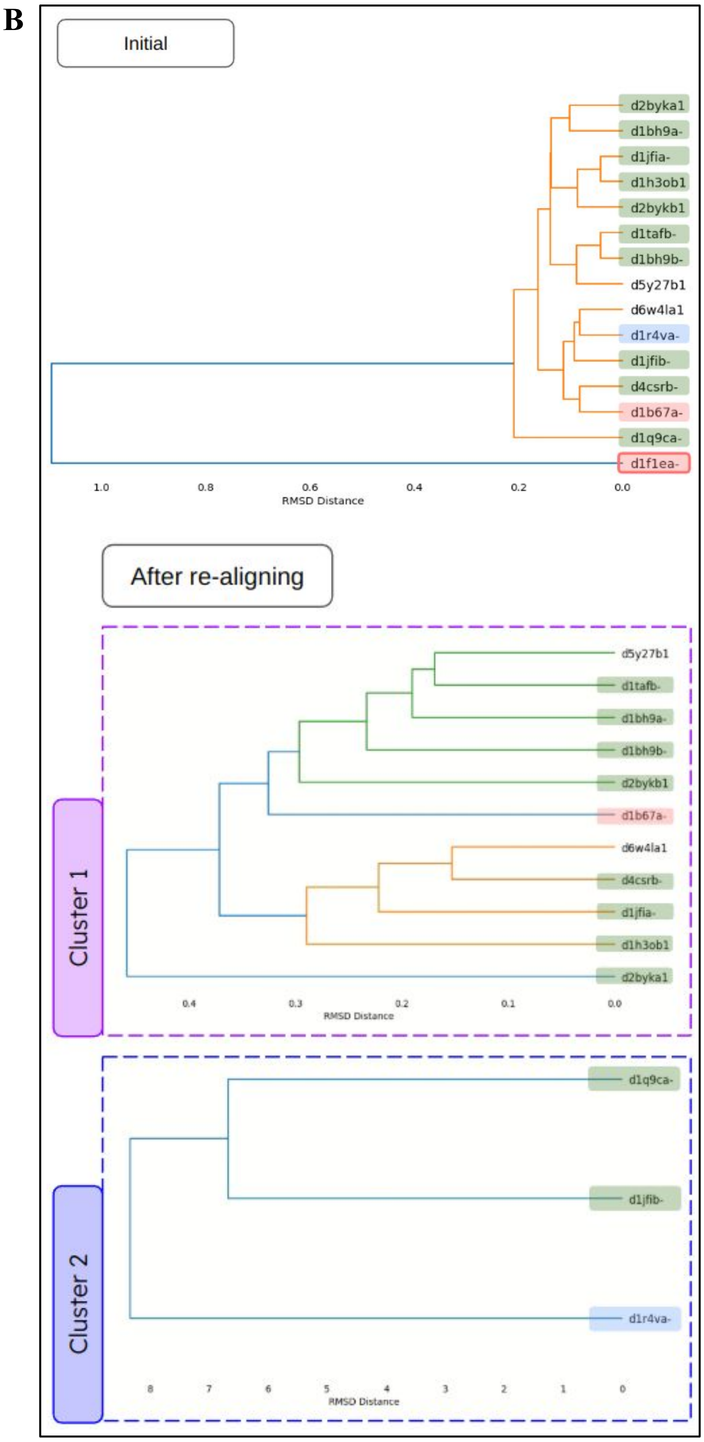

Supplementary Figure 4.

SCOPe superfamily 47113: Histone-fold superfamily. ***(A)*** *Domain classification according to SCOPe 2.08. The colour coding for the families presented here is followed all through the figure panels. The outlier is presented in read text, and wherever there is a box around the outlier label, the box has a red border.* ***(B)*** *RMSD-based dendrogram generated initially, and after the domains are divided into clusters and re-aligned. Note that the outlier is removed for re-alignment.* ***(C)*** *The topological diagrams of the domains generated using PDBsum1, labels coloured by family, grouped based on the results of the k-means clustering.*

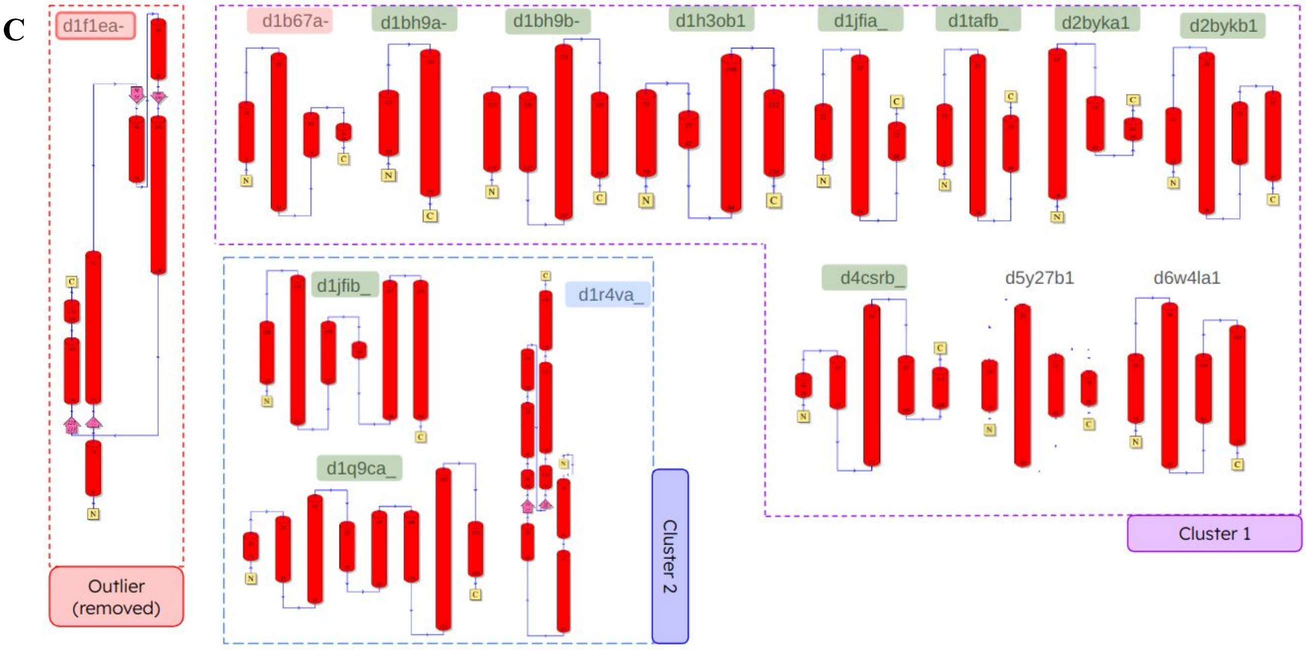

Supplementary Figure 5

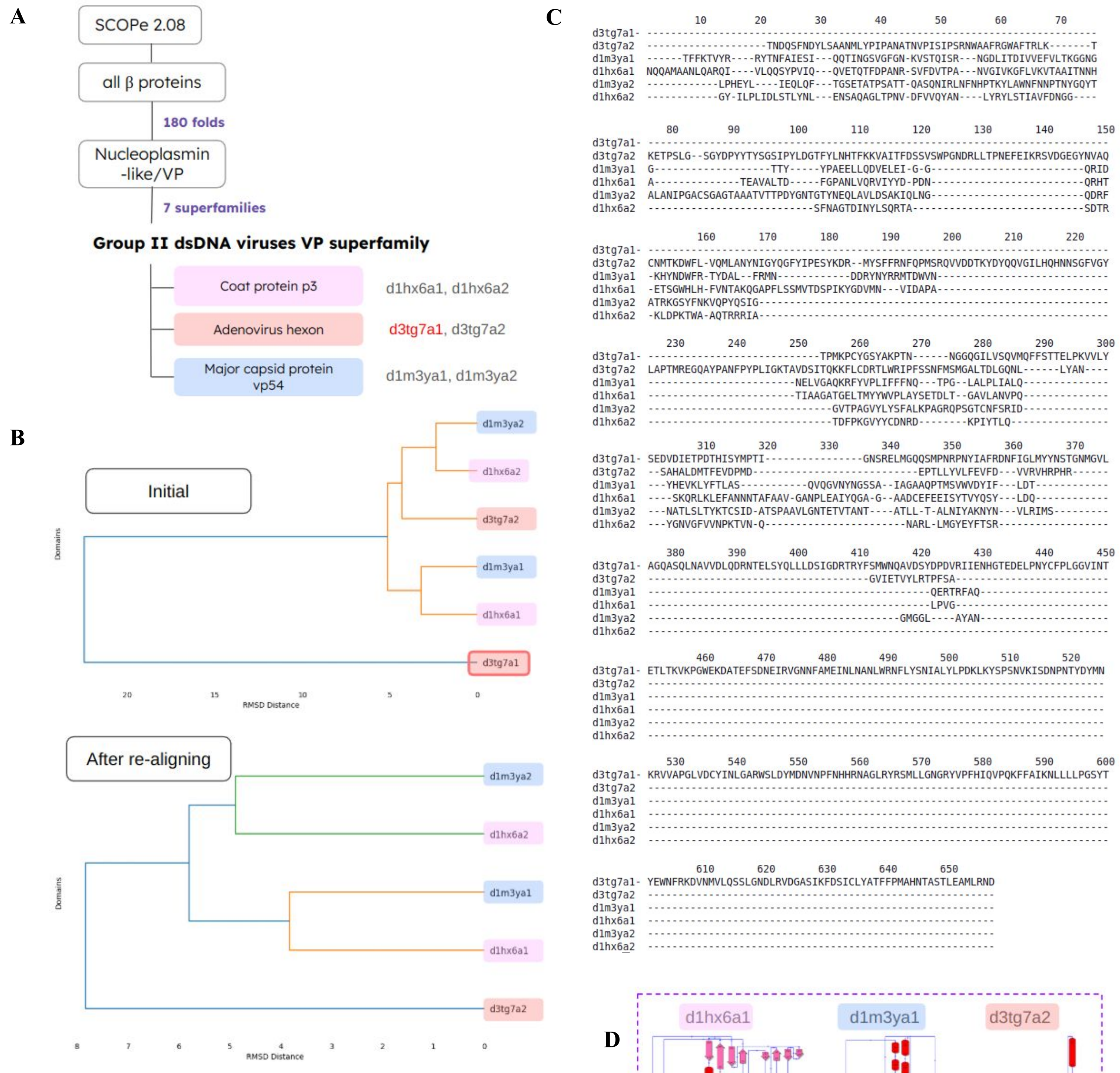

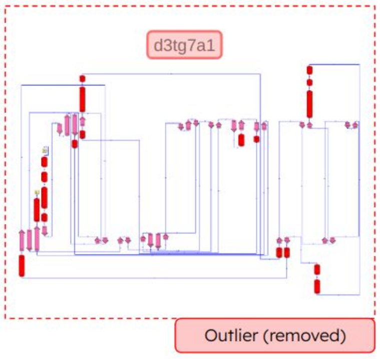

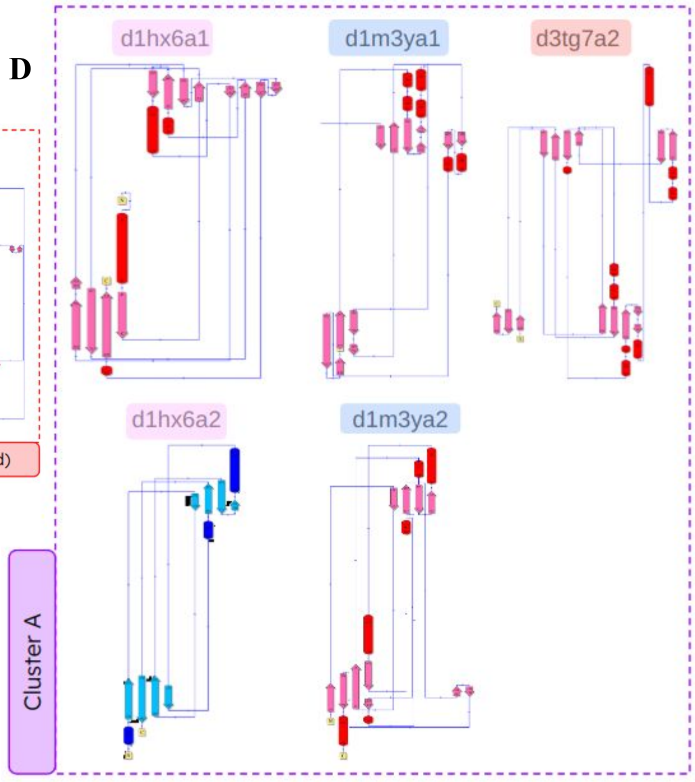

Supplementary Figure 5.
SCOPe superfamily 49749: Group II dsDNA viruses VP superfamily. ***(A)*** *Domain classification based on SCOPe 2.08. The colour coding for the families presented here is followed all through the figure panels. The outlier is presented in read text, and wherever there is a box around the outlier label, the box has a red border.* ***(B)*** *RMSD-based dendrogram generated initially, and after the domains are divided into clusters and re-aligned. Note that the outlier is removed for re-alignment.* ***(C)*** *The gappy alignment produced by removing only the terminal insertions of the outlier domain.* ***(D)*** *The topological diagrams of the domains generated using PDBsum1, labels coloured by family, grouped based on the results of the k-means clustering.*

Supplementary Figure 6

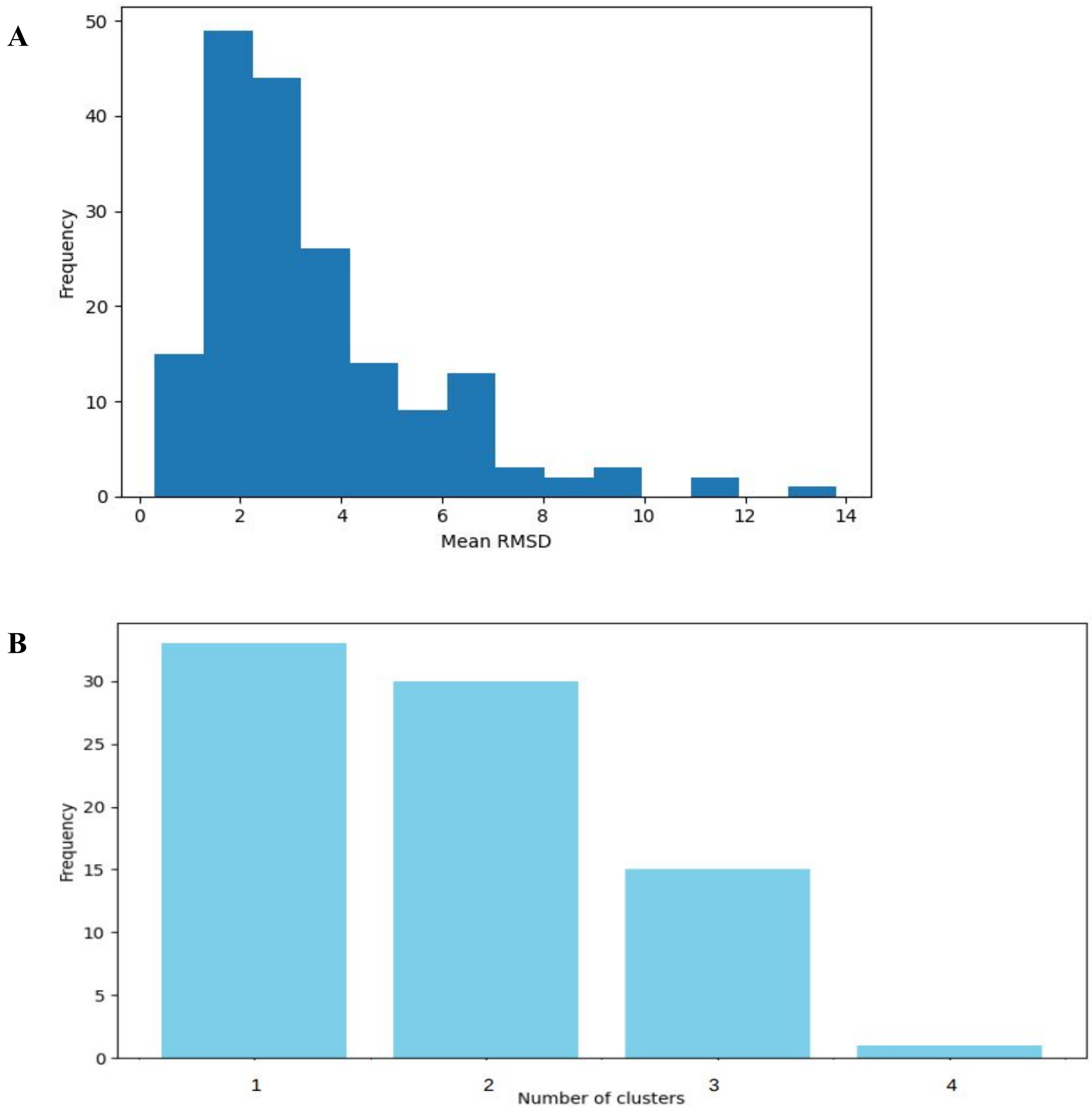

Supplementary Figure 6. *(A) Frequency distribution of the mean root mean square deviation (RMSD; in angstrom) of split superfamilies in PASS2.8. (B) Frequency plot of the number of clusters found for the 79 structurally divergent superfamilies in PASS2.8.*
