# Supplementary material for: PASS2: update of database of structure-based sequence alignments
Source: Database (Oxford). 2025 Nov 13;2025:baaf072. doi: 10.1093/database/baaf072 (PMC12612674; doi:10.1093/database/baaf072)
Supplement: baaf072_Supplemental_Files [file baaf072_supplemental_files.zip › PASS2_Suppl_table.pdf]

ADDITIONAL_FILE_2

TABLE S1: The initial average gap percentage, the improved average gap percentage, and the difference between these two values for all 79 superfamilies that presented problematic cases. The superfamilies are arranged by class and within each class, sorted by the difference in average gap percentage (descending order).

Class	Superfamily	Inital_Average_Gap_Percent	Improved_Average_Gap_Percent	Difference
All alpha	47781	62.03	23.32	38.71
All alpha	48600	58.22	19.68	38.54
All alpha	46955	48.57	27.58	21.00
All alpha	46894	44.54	23.71	20.83
All alpha	109604	57.03	42.03	15.00
All alpha	47113	48.13	36.26	11.87
All beta	50447	70.76	33.67	37.09
All beta	49785	72.57	36.69	35.88
All beta	51011	71.37	38.33	33.04
All beta	63380	62.08	30.61	31.47
All beta	50022	53.25	21.80	31.45
All beta	50939	54.95	25.67	29.28
All beta	50814	67.66	38.95	28.70
All beta	50692	51.17	22.65	28.52
All beta	49818	44.74	17.43	27.32
All beta	54160	47.47	21.76	25.71
All beta	50044	57.82	36.11	21.71
All beta	63748	52.15	33.05	19.11
All beta	49562	47.93	30.32	17.61
All beta	51283	44.14	28.53	15.61
All beta	49401	50.90	36.18	14.72
All beta	49749	55.82	41.19	14.63
All beta	49417	48.63	38.84	9.79
All beta	49265	55.62	46.54	9.09
Alpha and beta (a/b)	53223	56.16	19.15	37.02
Alpha and beta (a/b)	53659	61.88	24.90	36.98
Alpha and beta (a/b)	53671	56.84	20.66	36.18
Alpha and beta (a/b)	53448	67.51	31.47	36.04
Alpha and beta (a/b)	51556	66.94	33.19	33.75
Alpha and beta (a/b)	52440	64.81	32.79	32.03
Alpha and beta (a/b)	75217	51.27	21.42	29.85
Alpha and beta (a/b)	52467	62.09	32.73	29.36
Alpha and beta (a/b)	52777	54.75	25.97	28.78
Alpha and beta (a/b)	102405	47.46	19.87	27.60
Alpha and beta (a/b)	52317	69.17	42.38	26.79
Alpha and beta (a/b)	51569	63.97	38.47	25.50
Alpha and beta (a/b)	53756	64.34	38.99	25.35
Alpha and beta (a/b)	52518	63.55	39.03	24.52
Alpha and beta (a/b)	53613	46.83	22.89	23.93
Alpha and beta (a/b)	52283	47.43	24.80	22.64
Alpha and beta (a/b)	52374	70.92	49.87	21.05
Alpha and beta (a/b)	52266	56.53	37.14	19.39
Alpha and beta (a/b)	52799	58.20	38.94	19.26
Alpha and beta (a/b)	53254	49.83	31.88	17.96
Alpha and beta (a/b)	53927	65.74	48.06	17.68
Alpha and beta (a/b)	53271	54.77	37.11	17.66
Alpha and beta (a/b)	51395	61.30	46.22	15.08
Alpha and beta (a/b)	51621	65.73	50.70	15.03
Alpha and beta (a/b)	53383	58.17	44.75	13.42
Alpha and beta (a/b)	53697	52.15	39.05	13.11
Alpha and beta (a/b)	53901	57.62	47.64	9.98
Alpha and beta (a/b)	51366	48.06	42.19	5.88
Alpha and beta (a+b)	55961	61.02	24.67	36.35
Alpha and beta (a+b)	54189	47.44	11.61	35.83
Alpha and beta (a+b)	55909	55.02	19.70	35.32
Alpha and beta (a+b)	54534	69.95	34.65	35.30

Alpha and beta (a+b)	54373	62.25	31.20	31.05
Alpha and beta (a+b)	54236	63.37	33.66	29.71
Alpha and beta (a+b)	81301	59.08	31.86	27.22
Alpha and beta (a+b)	117856	42.42	15.51	26.91
Alpha and beta (a+b)	54593	60.90	35.47	25.43
Alpha and beta (a+b)	54292	49.96	29.39	20.57
Alpha and beta (a+b)	55469	49.44	28.93	20.51
Alpha and beta (a+b)	56300	55.76	36.83	18.93
Alpha and beta (a+b)	55174	59.60	40.76	18.84
Alpha and beta (a+b)	55874	45.78	27.95	17.83
Alpha and beta (a+b)	55331	36.72	20.37	16.35
Alpha and beta (a+b)	55550	49.53	33.91	15.62
Alpha and beta (a+b)	56399	54.59	40.95	13.65
Alpha and beta (a+b)	54523	45.26	31.82	13.44
Alpha and beta (a+b)	56024	49.72	39.03	10.69
Alpha and beta (a+b)	55681	60.38	50.23	10.15
Alpha and beta (a+b)	54495	44.78	37.45	7.33
Membrane and cell surface proteins and peptides	56935	40.62	27.06	13.56
Multi-domain proteins (alpha and beta)	56601	63.02	32.60	30.43
Small proteins	57501	61.50	30.66	30.84
Small proteins	57903	57.96	30.80	27.16
Small proteins	57586	46.60	27.79	18.81
Small proteins	57095	53.43	37.66	15.77
